# *In vitro* preclinical modeling of sex-specific biology and hormonal dynamics in women's health: insights across multiple organs

**DOI:** 10.3389/fgwh.2026.1878598

**Published:** 2026-06-30

**Authors:** Shiyan Tang, Jie Ji, Aakanksha Gulati, Yuncheng Man, Chaitra Belgur, Abidemi Junaid, Donald E. Ingber

**Affiliations:** 1 Wyss Institute for Biologically Inspired Engineering at Harvard University, Boston, MA, United States; 2 Institute of Environmental Medicine, Karolinska Institutet, Stockholm, Sweden; 3 Vascular Biology Program and Department of Surgery, Boston Children’s Hospital and Harvard Medical School, Boston, MA, United States; 4 Harvard John A. Paulson School of Engineering and Applied Sciences, Cambridge, MA, United States

**Keywords:** hormone dynamics, microphysiological systems (MPS), new approach methodologies (NAMs), organoid, organ-on-a-chip, sex as a biological variable (SABV)

## Abstract

Hormonal fluctuations in women have major impacts on organ physiology, contributing to well-recognized sex differences in disease susceptibility and manifestations, as well as therapeutic responses. Yet, most experimental studies and preclinical models fail to take sex-specific differences in tissue responses or changes in female hormones into consideration. In this Minireview, we highlight the importance of incorporating hormonal dynamics in experimental design and focus on emerging *in vitro* new approach methodologies (NAMs), including primary human cell cultures, organoids, and microfluidic human organ-on-a-chip (Organ Chip) models, which are being used to study hormone-dependent biology across multiple organ systems in a more clinically relevant way. Clinical data linking estrogen and progesterone signaling to sex-specific disease manifestations and the limitations of association-based studies are first reviewed. Then we describe how new human-relevant NAMs that can replicate female hormone-dependent exposures and responses *in vitro* may be used to dissect disease mechanisms and discuss each model's unique advantages and disadvantages. We also consider how these new approaches may be used as preclinical models for drug development, toxicity testing, and personalized medicine in the future.

## 1 Introduction

Women experience dynamic hormonal fluctuations—spanning the menstrual cycle, pregnancy, and menopause—where primary sex hormones, such as estrogen (17*β*-estradiol, E2) and progesterone (P4), exert wide-reaching effects far beyond the reproductive tract. Epidemiological evidence consistently demonstrates that these hormones shape disease risk, severity, and treatment response across virtually every organ system. For instance, in the lung, cyclical hormonal changes influence asthma severity and drive the “gender gap” in cystic fibrosis by modulating airway inflammation and mucociliary clearance (1–7). Similarly, sex hormones modulate intestinal barrier function in inflammatory bowel disease (IBD) (8–11), and dictate cardiovascular risk, where premenopausal estrogen confers endothelial nitric oxide synthase (eNOS)-mediated vasoprotection that is lost post-menopause (12–14). Liver metabolism and reproductive disorders like polycystic ovary syndrome (PCOS) and endometriosis are likewise profoundly driven by sexually dimorphic hormonal profiles (15–17).

**Figure 1:**
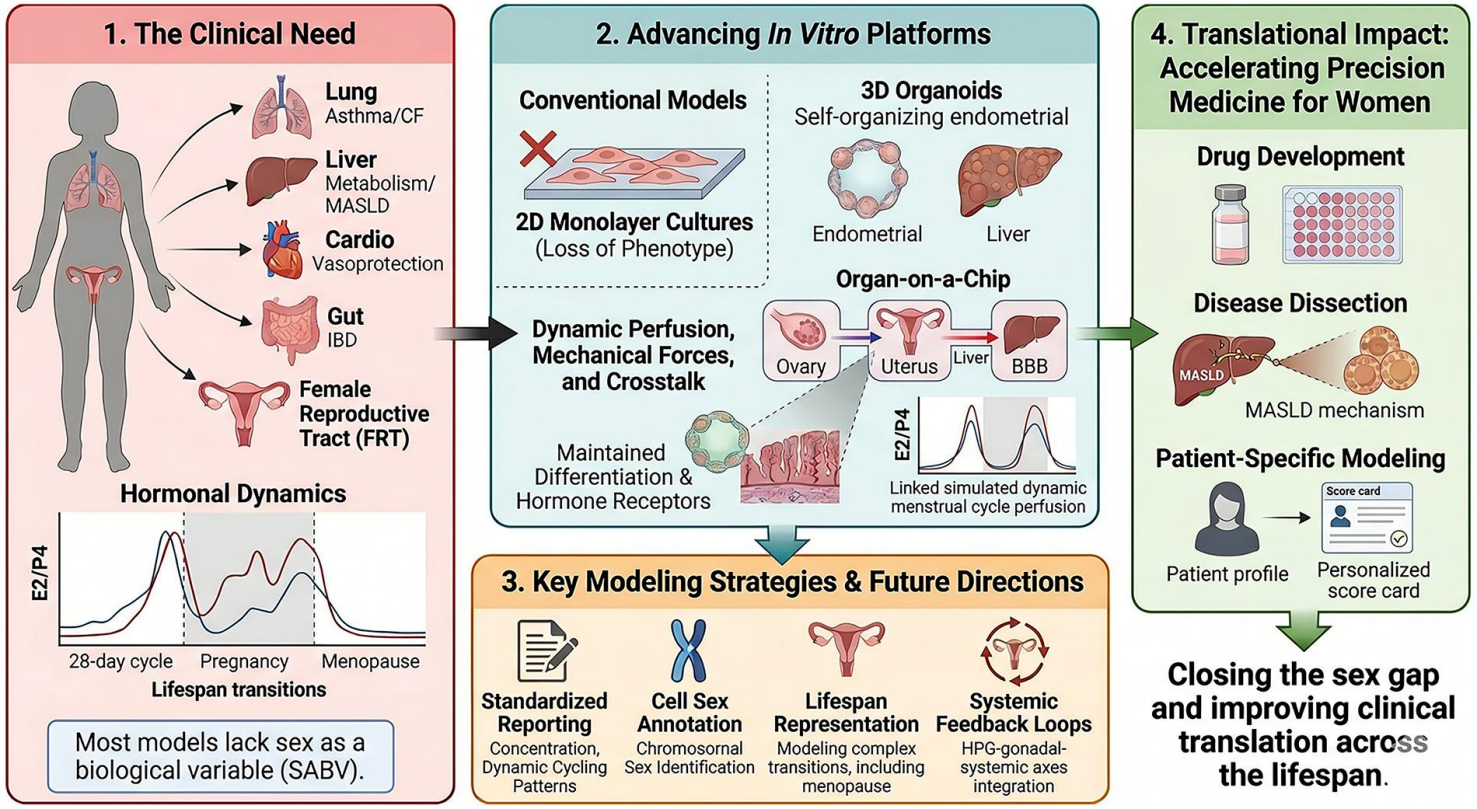
**Summary of hormone-aware *in vitro* modeling in women's health: From clinical context to translational solutions.** Schematic visualizing the workflow to bridge epidemiological gaps with advanced preclinical bioengineered NAMs. The narrative flows clockwise across four shaded quadrants: **(1) Clinical Need:** The problem is defined by dynamic systemic Estradiol (E2) and Progesterone (P4) fluctuations across lifespan transitions (28-day cycle, pregnancy, menopause), which govern pathophysiology across multiple organs (e.g., Lungs, Liver, Cardiovascular, female reproductive organs) yet are rarely incorporated into static, sex-agnostic models (left). **(2) Advancing *In Vitro* Platforms:** Advanced NAMs—specifically 3D Organoids (retaining differentiation and receptor expression) and multi-organ Organ-on-a-Chip (incorporating microfluidic flow, mechanical cues, immune cells, multi-organ crosstalk, and dynamic rolling 28-day E2/P4 menstrual cycle simulation)—overcome traditional limitations (center top). **(3) Key Modeling Strategies & Future Directions:** Methodology requirements for standardization include Standardized Reporting (concentration/cycling), mandatory Cell Sex Annotation (XX/XY tracking), and dynamic Lifespan Representation (bottom center). **(4) Translational Impact**: These hormone-aware models can accelerate precision medicine by enabling predictive Drug Development, accurate Disease Mechanism Dissection (e.g., MASLD in spheres), and Patient-Specific Modeling (e.g., personalized receptivity scoring; right). The ultimate goal is closing the preclinical sex gap and optimizing women's health interventions across the entire lifespan. Diagram created with Gemini Nano Banana Pro.

Despite this clear clinical impact, the mechanistic underpinnings of hormone-dependent pathophysiology remain poorly understood (18). Clinical association studies cannot definitively establish causality or isolate cellular mechanisms, while traditional animal models lack translational relevance due to divergent reproductive cycles and reproductive anatomy. Consequently, there is a pressing need for controlled, human cell-based *in vitro* systems. Propelled by recent Food and Drug Administration (FDA) (19) and National Institutes of Health (NIH) initiatives (20), the field is rapidly adopting new approach methodologies (NAMs), such as human organoids and microfluidic human organ-on-a-chip (Organ Chip), to improve clinical translation (21). However, as these advanced platforms are embraced, a critical variable is frequently overlooked: the dynamic nature of female endocrinology. To fully realize the potential of NAMs, it is insufficient to engineer hormonally static or sex-agnostic models; experimental designs must integrate sex-specific biology and physiological hormonal cycling as well.

In this Minireview, we evaluate the capacity of emerging *in vitro* platforms, including two-dimensional (2D) cultures, three-dimensional (3D) organoids, and microfluidic Organ Chip, to model hormone-dependent biology across multiple human organ systems. We highlight key advances, compare model strengths and limitations, and discuss their translational implications for drug development, toxicity testing, and personalized medicine, ultimately proposing a framework for a more hormone-aware approach to preclinical research that will be more relevant for women's health (Figure 1).

A literature search was conducted across PubMed and Web of Science for articles published between 2014 and 2026, using key terms such as “Sex as a Biological Variable (SABV),” “hormone dynamics,” “Organ-on-a-Chip,” “organoids,” and “Microphysiological Systems.” Article inclusion was strictly limited to peer-reviewed, English-language studies employing human-relevant NAMs to model sex-specific disease mechanisms, endocrine responses, and pharmacological outcomes across multiple organ systems.

## 2 *In vitro* models to study female hormone effects across organs

### 2.1 Conventional 2D culture models

Early *in vitro* studies of hormone effects on cellular behavior relied almost exclusively on use of conventional 2D cell monolayer cultures. While analysis of hormone-responsive cells (e.g., endothelial cells exposed to estrogen, endometrial fibroblasts decidualized with progesterone) in standard planar culture plates has provided valuable baseline mechanistic insights, they are fundamentally insufficient for understanding systemic hormone effects that rely on complex tissue-tissue interactions and local microenvironmental cues experienced *in vivo*. Planar cell monolayers lack the complex 3D tissue architecture, extracellular matrix (ECM), mechanical cues, and multi-cellular crosstalk required to maintain proper cell polarity and paracrine signaling (22–24). Cells grown on rigid plastic substrates might respond to a hormone initially, but they often rapidly lose their tissue-specific differentiated phenotype outside of their native tissue context (25). For example, primary hepatocytes in 2D culture dedifferentiate within days: they become flattened, reduce the expression of liver-specific genes, own-regulate key detoxification enzymes, secrete significantly less albumin, and cease many other specialized functions, reflecting a rapid collapse of their *in vivo* functionality (23, 26, 27). Similarly, vaginal epithelial cells cultured in 2D often fail to recapitulate the expression of mucin proteins, junctional markers, and surface antigens observed in 3D models and native tissues (28). Consistent with these functional differences, transcriptomic profiling identified over 1,000 differentially expressed genes between 2D and 3D cultures of the same Fallopian tube secretory epithelial cells (29). These molecular discrepancies translate into poor predictive validity, as 2D-derived findings frequently fail to recapitulate *in vivo* cervical cancer responses (30).

Because conventional planar cultures often fail to maintain stable hormone receptor expression or downstream signaling pathways over time, they cannot accurately model how hormones act on a complete tissue microenvironment. While they can be useful for highly reductionist assays, the failure of conventional monolayers to preserve the complex 3D microenvironment necessary for authentic, sustained hormone responses has driven the development of advanced 3D organoid and microfluidic platforms.

### 2.2 Organoids and 3D engineered tissue models

The advent of organoid technology has substantially advanced the capacity to study sex-specific biology *in vitro*. Organoids are self-organizing 3D structures created by culturing stem cells isolated from primary tissues or induced pluripotent stem (iPS) cells within a 3D ECM gel (e.g., Matrigel), which recapitulate many architectural and functional features of their tissue or organ of origin (31). Critically, when cultured in this manner, organoids are often able to maintain hormone receptor expression and responsiveness over extended culture periods, directly addressing the most significant limitation of 2D cultures systems. Today, organoids serve as powerful platforms for modeling biology in a hormone-dependent context across multiple tissue and organ systems (32–35).

#### 2.2.1 Hepatic models: metabolism and disease

In the liver, sex differences arise from the interplay between cell-intrinsic sex-chromosome programs and circulating gonadal steroids acting through nuclear receptors, such as estrogen receptor *α* (ER*α*) and androgen receptors (ARs). This signaling tunes lipid uptake, *β*-oxidation, and fibrogenic cascades (17, 36). Direct experimental support comes from two complementary primary human hepatocytes (PHHs) studies (37, 38). At baseline, male PHHs exhibit higher low density lipoprotein receptor (LDLR) mRNA levels than female cells, and physiological 17*β*-estradiol, testosterone, or progesterone induced sex-specific shifts in lipid metabolism genes — estradiol selectively regulated genes (PPARA, LIPC, and APOL2) only in female PHHs, while ABCA1 and APOA5 responses were both sex- and hormone-dependent (37). Critically, rapid hormone metabolism within the culture period highlights a key limitation of static PHH models, underscoring the need for perfused systems to maintain physiological endocrine states. Under steatotic conditions, sex differences were amplified: female PHHs showed superior lipid excretion via enhanced very-low-density lipoprotein (VLDL) secretion, while male PHHs mounted stronger transcriptional responses (38). Collectively, these studies establish donor sex and hormonal context as independent determinants of hepatocellular lipid metabolism, demonstrating that PHH-based NAMs can capture clinically relevant metabolic dysfunction-associated steatotic liver disease (MASLD) sex differences only when hormonal dynamics are explicitly incorporated.

Furthermore, sex-steroid preconditioning of engineered iPS cell-derived multi-lineage liver spheres were (estradiol for female spheres, testosterone for male) were shown to modulated disease-relevant steatosis- and fibrosis-like transcriptional programs when benchmarked against clinical MASLD/ metabolic dysfunction–associated steatohepatitis (MASH) datasets (39). Single-nucleus RNA-seq further identified a proliferative hepatocyte state with cancer marker expression under estradiol conditions, highlighting potential risks of hormone-based therapies. Complementing this, multilineage spheroids were used to elucidate female-specific susceptibility to fatty liver disease, demonstrating that ER*α* directly drives lipid accumulation by upregulating the PNPLA3 p.I148M variant utilized (40). Together, these models align with *in vivo* evidence that estrogen and androgens critically reprogram hepatocyte metabolism and regeneration (41).

#### 2.2.2 Renal models: physiology and function

Advanced 3D kidney organoids, established from adult human tissues or iPSCs, can now faithfully recapitulate human renal architecture (42–46) and have been extensively utilized to model the pathogenesis such as polycystic kidney disease (PKD) (47). Despite their emergence as robust tools for studying renal biology and pathophysiology, the field has been slower to incorporate sex as a biological variable. Consequently, there has been a recent call to action arguing that sex-specific differences in renal function, drug handling, and disease susceptibility demand the systematic incorporation of chromosomal sex and hormone signaling into engineered models (48).

#### 2.2.3 Mammary models: breast biology and disease

Over the past decade, human breast organoids have emerged as powerful platforms for studying sex hormone biology across normal physiology and disease. In normal breast tissue, organoids retain functional ER and PR expression and recapitulate hormone-driven epithelial responses, with single-cell profiling resolving how estrogen, progesterone, and prolactin collectively remodel distinct luminal and basal cell populations (49–51). These hormone-competent systems have been extended to disease modeling: benign organoids from BRCA1 carriers exposed to a simulated menstrual hormone cycle demonstrated that germline mutations rewire PR signaling and alter hormone-dependent cancer risk (52, 53); patient-derived tumor organoid biobanks retained ER/PR/HER2 status across subtypes and enabled clinically concordant endocrine drug screening (49, 54, 55); specialized media engineering and suspension culture resolved the longstanding barrier of long-term ER maintenance (50, 56); and co-culture with cancer-associated fibroblasts uncovered stromal paracrine drivers of endocrine resistance (57). These advances position human breast organoids as uniquely tractable systems for uncovering how hormonal context — across the menstrual cycle, pregnancy, and disease — shapes epithelial cell fate and drug response, opening new avenues for precision endocrine therapy and sex-informed preclinical modeling.

#### 2.2.4 Reproductive models: female reproductive tract and placenta

Organoid technology has enabled systematic *in vitro* reconstruction of the hormone-dependent biology of the female reproductive tract, from gamete transport to placentation. In the endometrium, multiple independent groups have established long-term, expandable epithelial organoids that faithfully recapitulate the estrogen- and progesterone-driven transitions of the menstrual cycle — differentiating into secretory and ciliated cell types while maintaining ER*α*, PR, mucin expression, and glandular architecture characteristic of the mid-secretory phase (35, 58–61). Transcriptomic profiling across these models consistently demonstrated that progesterone directly attenuates estrogen-driven proliferative programs, recapitulating the hormonal antagonism that governs endometrial receptivity *in vivo*. Decidualization — the progesterone-dependent stromal transformation essential for embryo acceptance — has been further replicated using human chorionic gonadotropin (hCG) and prolactin stimulation (58), while androgen excess in a scaffold-free multicellular endometrial organoid model recapitulated PCOS-associated endometrial dysregulation (62), underscoring the sensitivity of these systems to diverse hormonal inputs.

Moving toward the embryo-maternal interface, increasingly complex 3D co-culture models have been developed. A stromal-epithelial composite organoid successfully mimicked day-14 endometrial receptivity (63), and an embryo-endometrial co-culture system recapitulated yolk sac formation and trophoblast invasion at the implantation site (64) — events that are otherwise inaccessible to direct study in humans due to ethical constraints. Complementing these uterine models, trophoblast organoids self-organize into villous-like structures and constitutively secrete placental hormones including hCG and placental lactogen, making them a uniquely powerful NAM for studying the placenta as an active endocrine organ during early pregnancy (32). Finally, at the proximal end of the reproductive tract, fallopian tube organoids derived from bipotent epithelial stem cells generate both ciliated and secretory cell types and modulate ciliary beat frequency and secretory output in response to physiological estradiol and progesterone (65–67). These models enable mechanistic dissection of hormone-dependent gamete transport — a process whose dysfunction underlies both infertility and ectopic pregnancy — in a fully human, tractable *in vitro* system.

### 2.3 Microfluidic organ-on-a-chip

While organoids excel at establishing local 3D tissue architecture, they generaly lack tissue-tissue interfaces, immune cells, and the biophysical cues associated with mechanical motions (e.g., cervical and uterine contraction) and dynamic vascular perfusion (e.g., fluid shear stress) required to fully mimic organ- and system-level physiology. Organ Chip technology bridges this gap. Representing the most sophisticated class of *in vitro* NAMs, Organ Chip combine the ability to precisely recreate tissue-tissue interfaces and integrate circulating or tissue-resident immune cells with precise microfluidic and mechanical control, while also enabling the delivery of hormones in time-varying, physiologically realistic patterns and even support multi-organ communication via fluidic coupling (68–70).

#### 2.3.1 Gastro intestinal and hepatic systems

Intestine Chips lined by organoid-derived epithelium under dynamic fluid flow recapitulate villus-like morphogenesis, multi-lineage differentiation, and mucus production, with transcriptomic profiles more closely resembling *in vivo* human intestine than organoids alone (71, 72), and the addition of physiologically relevant oxygen gradients further enables co-culture with complex communities of aerobic and anaerobic commensal bacteria directly in contact with the overlying mucus layer (73, 74). These capabilities have enabled modeling of diverse intestinal diseases, including radiation-induced barrier injury for drug testing (75), species-specific enhancement of enterohemorrhagic *E. coli* pathogenesis by human microbiome metabolites (76), enteric SARS-CoV-2 infection (77) and human Colon Chips lined by IBD patient-derived colonic epithelium interfaced with matched stromal fibroblasts (78). Strikingly, perfusion of female-derived IBD Chips with pregnancy-associated hormones (E2, MPA, hCG, prolactin, placental lactogen) markedly exacerbated the IBD phenotype, including increased cytokine production and stromal fibrosis, directly recapitulating the pregnancy-associated flares seen clinically in women with IBD and powerfully demonstrating how Organ Chip technology captures hormone-driven, sex-specific responses that organoids and animal models cannot (78). Together, these findings establish a compelling precedent for integrating sex hormone biology into intestinal Organ Chip platforms, opening new avenues for dissecting how gonadal steroids regulate gut physiology and disease susceptibility in women.

Human Liver Chips incorporating primary hepatocytes alongside sinusoidal endothelial cells, Kupffer cells, and hepatic stellate cells under physiological flow detect diverse phenotypes of drug-induced liver injury and clinically relevant species-specific toxicities (79), while significant inter-donor variability in hepatic drug metabolism between female and male donors underscores sex as an independent variable in pharmacokinetic modeling (80). A microfluidic model of the liver acinus further revealed that metastatic breast cancer growth and resistance to estrogen deprivation are governed by local estradiol concentrations and oxygen gradients within the liver microenvironment — a spatially nuanced hormonal interaction inaccessible to conventional culture systems (81). Together, these findings underscore sex and sex hormones as critical variables in liver chip research, pointing toward hormone-integrated, sex-matched platforms as essential tools for modeling sexually dimorphic hepatic physiology and disease.

#### 2.3.2 Vascular microenvironments and endothelial dynamics

Blood Vessel Chips also have been developed and widely used to investigate endothelial cell responses, though few have isolated direct sex hormone effects. One study addressed this using a microfluidic device in which estradiol inhibited ATP release from erythrocytes, thereby reducing ATP-stimulated nitric oxide production in adjacent endothelial cells — revealing a novel hormone-vascular crosstalk mechanism relevant to estrogen therapy-associated thrombosis risk (14). Complementing this, conventional culture studies have established that biological sex is itself an intrinsic determinant of endothelial phenotype: female endothelial cells exhibit higher basal eNOS activity and nitric oxide production than male counterparts (82), display sex- and tissue-specific differences in redox status and inflammatory responses (83), and respond divergently to shear stress and substrate stiffness through sexually dimorphic YAP1 mechanosensing (84). The profound intrinsic sex differences in endothelial biology revealed by these studies make a compelling case for incorporating donor sex and physiological hormone levels as standard variables in next-generation Blood Vessel Chip models.

#### 2.3.3 Female reproductive tract microphysiology

The organs of the female reproduct tract are arguably the most extensively and successfully modeled system using microfluidic Organ Chip technology. The profound effect of estradiol on epithelial differentiation has been demonstrated in a human Vagina Chip, where 4 nM *β*-estradiol induced significantly greater differentiation (upregulating ER*α*, PGR, PCK1, and ZO-1) compared to 0.4 nM concentrations (85). Cervix Chips have modeled human cervical biology under high-E2 vs. high-P4 states, revealing hormone-specific patterns of mucus production, barrier function, and innate immunity (86, 87). Similarly, Fallopian Tube Chips exposed to high E2 vs. high P4 hormonal conditions that mimic the follicular and luteal phases demonstrate distinct ciliary and secretory responses (88), while elevated testosterone in these systems alters cilia beating consistent with PCOS pathophysiology (89).

#### 2.3.4 Endometrial receptivity and multi-organ integration

The endometrium has been modeled with direct relevance to personalized reproductive medicine. A dual-chamber microfluidic model that co-cultures primary endometrial stromal fibroblasts with endothelial cells under a simulated 28-day menstrual cycle was shown to undergo *in vivo*-like morphological decidualization (90). A follow-up study demonstrated that hemodynamic shear forces actually *enhanced* stromal decidualization via endothelial-derived prostaglandins—a mechanically-sensitive hormonal crosstalk impossible to observe in static culture (91). A vascularized Endometrium Chip also was used to evaluate patient-specific endometrial receptivity, creating an actionable scoring system integrating molecular profiling with quantitative angiogenesis (92). An Ovary-Endometrium Chip also was developed with biomarker-driven feedback to model paracrine crosstalk to move toward systemic modeling (93).

Perhaps the most ambitious demonstration of hormone-aware bioengineering is the multi-organ menstrual cycle platform (94). This system integrated ovary explants with fallopian tube, uterus, cervix, and liver culture modules via microfluidic channels, dynamically cycling hormones to replicate a 28-day menstrual cycle. Impressively, although the different organ modules were not all lined by human cells, the cultured ovary explants recapitulated ovarian follicle growth, maturation, ovulation, and granulosa cell luteinization over a normal 28-day time scale. This multi-organ “Body-on-Chips” approach and others, such as the Quintet-MFP system, represent a paradigm shift in modeling systemic endocrinology (95–97).

### 2.4 Integrating sex as a biological variable in advanced models

Treating sex as a biological variable (SABV) is not a mere reporting formality; it is a fundamental experimental parameter that dictates cellular baseline states and violently biases response-to-injury or drug-exposure phenotypes (98). The NIH SABV policy now mandates that sex be factored into research design for vertebrate animal and human studies. In respiratory preclinical research, SABV was extended explicitly to *in vitro* lung models, recommending that investigators authenticate cellular sex, capture donor metadata (e.g., menopausal status, hormone therapy), and prevent inadvertent steroid receptor activation by using phenol red-free and charcoal-stripped medium (18). Supporting this, sex steroids have been shown to directly shape pulmonary epithelial phenotypes by modulating ion channel expression and airway surface liquid homeostasis (2, 99, 100).

Similarly, failing to incorporate SABV misses key sex-specific mechanisms and drug responses (48). Engineered organoids and Organ Chip provide highly controlled *in vitro* tools that can enable manipulation of these genetic, epigenetic, and hormonal factors—including timed hormone conditioning. Ultimately, incorporating systemic SABV into advanced bioengineered platforms is essential to improve the generalizability of preclinical studies, facilitate sex-stratified drug evaluation, and inform clinical trial design with respect to hormonal state.

### 2.5 Comparative advantages and limitations

Each of the *in vitro* model systems focused on here — 2D cultures, organoids, and microfluidic Organ Chip — offers distinct advantages and trade-offs for studying hormone-dependent biology (Table 1). Beyond platform-specific trade-offs, a critical limitation shared by all current *in vitro* systems is their inability to fully replicate systemic hormonal feedback loops, most notably the hypothalamic-pituitary-gonadal (HPG) axis that governs cyclic hormone production. While multi-organ platforms incorporating ovarian tissue have begun to address this, complete reconstitution of the complex HPG endocrine regulator*y* axis remains beyond current capabilities. Additionally, a key material consideration for microfluidic systems is the adsorption of hydrophobic compounds, including steroid hormones, by polydimethylsiloxane (PDMS) device materials. Recent studies show that aldosterone exhibits no detectable interaction with PDMS, estradiol displays moderate interaction, and progesterone shows strong interaction (101). However, many commercial Organ Chip systems composed of alternative, non-absorption plastics are now available. Finally, most studies fail to specify the baseline hormonal state of the primary cells used, making cross-study comparisons difficult and potentially introducing systematic bias into preclinical modeling.

**Table 1:** Comparison of *in vitro* models for modeling sex-specific biology. The table summarizes the methodological strengths and trade-offs of 2D monolayers, organoids, and Organ Chip systems, highlighting their respective capacities to replicate systemic hormonal dynamics, multi-organ crosstalk, and complex tissue microenvironments.

Model	Primary Advantages	Key Limitations
2D Cultures	Simplicity, high scalability, and ease of genetic manipulation. Compatible with general lab equipment.	Sacrifice 3D tissue architecture; frequently lose hormone receptor expression and phenotype over time.
Organoids	Restore 3D tissue organization, maintain differentiated phenotypes, and self-organize in response to hormone signals.	Typically lack vascular, stromal, and immune components; operate under static conditions that cannot replicate flow-dependent hormone delivery or cyclic menstrual dynamics.
Organ Chip	Greatest degree of physiological control, including flow, mechanical cues, tissue-tissue interfaces, immune cells, cyclic hormone delivery, and multi-organ communication, and preserved cell polarity at tissue interfaces.	Technically demanding, lower-throughput, and require specialized expertise and infrastructure.

## 3 Implications for treatment and therapeutic development

Hormonal status is a critical yet often overlooked modifier of drug efficacy, toxicity, and disease progression. As outlined earlier, variations in sex hormones can fundamentally alter pharmacokinetics, target tissue responsiveness, and systemic vascular signaling, contributing to well-documented sex differences in therapeutic benefit and adverse events (102–104). Accordingly, sex-specific dosing strategies and the consideration of a patient's endocrine state (e.g., menstrual cycle phase, menopause, or concurrent hormone therapy) represent practical avenues to improve treatment outcomes. The ability of microfluidic Organ Chip to mimic clinical drug pharmacokinetics by incorporating dynamic fluid flow (105) and the demonstration that this enhances predictive power for individual patients relative to static organoids (106), offers a new approach to personalized medicine that can benefit women as well as men. In parallel, the integration of the hormone-aware *in vitro* models discussed in this review—specifically 3D organoids and microfluidic Organ Chip — offers a scalable framework for screening these effects. By moving away from static 2D cultures and animal models, these advanced human-relevant platforms allow researchers to mechanistically dissect hormone-dependent drug responses. Together, these approaches support a vital shift toward more predictive preclinical testing and more precise, personalized therapeutic development.

## 4 Future directions

Advancing hormone-aware *in vitro* modeling requires addressing four critical priorities to fully realize the potential of these bioengineered platforms. First, the field urgently needs standardized reporting of *in vitro* hormonal conditions — specific concentrations, media formulations, and cycling patterns across 2D, 3D, and chip-based systems — to improve cross-study reproducibility. Second, systematically tracking and reporting donor cell chromosomal sex is essential to prevent confounding results, particularly in multi-organ platforms. This is a critical issue already highlighted by the kidney (48) and lung (18) modeling communities. Third, the next frontier involves leveraging the precise fluidic control of Organ Chip to integrate time-dependent, physiological hormone cycling. Building on foundational 28-day models (94), future systems must dynamically mimic complex endocrine transitions, including the historically underserved frontier of menopause. Finally, preclinical models must expand to encompass the entire spectrum of women's health across the lifespan, mimicking tissue responses from early pregnancy through post-menopause. Given the sustained underfunding of women's health research (107), developing and deploying these comprehensive preclinical tools is not just a scientific necessity, but an equity imperative.

## 5 Conclusion

Sex as a biological variable is no longer optional in preclinical research—it is a scientific imperative. The epidemiological and experimental evidence reviewed here demonstrates that hormonal dynamics exert profound effects on disease susceptibility and therapeutic responses across virtually every organ system, from the lung and liver to the cardiovascular system and the full spectrum of reproductive organs.

Advanced *in vitro* NAMs, and particularly the human organoid and Organ Chip platforms detailed in this review, are uniquely suited to dissect these systemic effects with mechanistic precision and human biological relevance. Yet, these tools remain underutilized in hormone-aware experimental designs, representing both a significant gap and an important scientific opportunity. The systematic incorporation of dynamic E2 and P4 cycling into human cell-based models, accurate sex annotation of cell sources, and expansion of multi-organ microfluidic platforms will collectively strengthen the translational value of preclinical research. Ultimately, this paradigm shift will accelerate the development of sex-informed therapeutics and advance the goal of precision medicine to improve the health of all women.
